# Parents’ and Professionals’ Perceptions on Causes and Treatment Options for Autism Spectrum Disorders (ASD) in a Multicultural Context on the Kenyan Coast

**DOI:** 10.1371/journal.pone.0132729

**Published:** 2015-08-12

**Authors:** Joseph K. Gona, Charles R. Newton, Kenneth Rimba, Rachel Mapenzi, Michael Kihara, Fons J. R. Van de Vijver, Amina Abubakar

**Affiliations:** 1 Centre for Geographic Medicine Research (Coast), Kenya Medical Research Institute, P.O. Box 230–80108, Kilifi, Kenya; 2 Department of Psychiatry, University of Oxford, Oxford, United Kingdom; 3 Psychology Department, United States International University-Africa, Nairobi, Kenya; 4 Department of Culture Studies, Tilburg University, Tilburg, Netherlands; 5 Department of Psychology, Lancaster University, Lancaster, United Kingdom; University of New South Wales, AUSTRALIA

## Abstract

**Objective:**

To explore parents’ and professionals’ perceived causes and treatment of Autism Spectrum Disorders (ASD) on the Kenyan Coast.

**Methods:**

In-depth interviews and focus group discussions using guiding questions were utilized in data collection. One hundred and three participants, who included parents of children with ASD, special needs teachers, clinicians, and social workers from diverse cultural background, participated in this study. The interviews and focus groups were recorded, transcribed verbatim and then translated to English. Themes were generated using content analysis.

**Results:**

Preternatural causes were mentioned and included evil spirits, witchcraft, and curses. Biomedical causes comprised infections, drug abuse, birth complications, malnutrition, and genetic related problems. Treatment varied from traditional and spiritual healing to modern treatment in health facilities, and included consultations with traditional healers, offering prayers to God, and visits to hospitals.

**Conclusions:**

The results suggest that regardless of cultural backgrounds, people on the Kenyan Coast have similar views on perceived causes and treatment of ASD. These findings provide valuable conceptual understanding for professionals when planning and implementing community based rehabilitation interventions targeting children with ASD within a local context.

## Introduction

The origin of autism spectrum disorders (ASD) is unknown; however, different cultural groups have their own perceptions of the causes of the disorder. Western biomedicine considers ASD as a neurodevelopmental disorder with a significant genetic contribution [1]. Cultural factors have recently been documented to influence characterization, diagnosis and treatment of ASD globally [2]. Because of its unique sociocultural environment, Africa may experience divergent conceptions on ASD compared to other cultures [3].

The role of religion in multi-cultural settings offers a major basis of support for families of children with ASD. In some cultures in the West, where majority of the people are born into Catholicism, mothers emulate Virgin Mary in accepting children with ASD through sacrifice and dedication and believed that the child was a sign from God [4]. Their religion gives them strength and patience. Other cultures have positive attitudes on ASD because of the strong faith such cultures have in their religion [5].

Parental beliefs about ASD diagnosis and treatment are influenced by their cultural background. Culture can be described as the values, beliefs, language, rituals, traditions, and other behaviors that are passed from one generation to another [6]. It is culture that forms individual and family beliefs about disability in general and ASD in particular [7].

There is no cure for ASD, however with early intervention and appropriate treatment and education, many people with ASD can function productively and attain some degree of independence. In the absence of conclusive information on the causes and treatment of ASD, parents of children with ASD develop their own perceptions of the disorder. Their interpretation of the etiology of ASD influences their judgment of the child and the effect the child has on the family [8]. If parents believe that the child’s disability is a punishment for sins committed, then they may have a negative view of the situation. Such views can negatively influence treatment seeking behaviors of parents of children with disabilities. Alternatively, if the disability is viewed as a blessing from God, more positive appraisals are expected. Parents’ beliefs on the nature and cause of disability provide the context for parents’ beliefs about intervention [9]. Their attitudes direct their actions and may have an effect on decision making about intervention [10] and future health care [11]. No study has attempted to explore the influence of local cultures on causes of ASD and how it affects treatment seeking behaviors in Kenya.

In order to offer support and guidance to parents in making help seeking decisions for their child living with ASD, it is essential to understand parents’ and professionals’ perceptions on the causes of ASD and what types of treatment parents seek.

### Research questions

1. What are the perceived causes of ASD on the Kenyan Coast from a parental and professional perspective?
2. What do parents and professionals in these settings perceive as treatment options and expectations for children living with ASD?

## Methodology

This study utilized a qualitative design and applied a phenomenological methodology guided by the “Explanatory Model” framework as described by Kleinman et al. [12]. This model explains the beliefs one holds about an illness or disorder, the personal and social meaning attached to the disorder and expectations about what will happen.

### Study site

The study was conducted on the Kenyan coast utilizing two counties with different cultures: Kilifi and Mombasa. Kilifi is predominantly occupied by the nine Mijikenda sub-groups (90%) and has a poverty level of 68% [13]. About 70% of the people are Christians, 20% practice traditional religions and about 10% belong to the Islam. Mombasa is mostly (80%) Muslim dominated area with people of Arab-culture constituting the majority of the population. It has a poverty level of 18% [14]. A number of churches exist for believers in Christian faith which constitutes about 15% of the population. Other religious groups, including Hindus and Buddhists, make up about 5% of the population.

### Study participants

Study participants included in this study were parents of children with a presumptive diagnosis of ASD and professionals. Participants were from different religious beliefs and included Christians, Muslims, and traditionalists, which represents the main communities in the two counties. The educational levels of parents from both settings ranged from no formal schooling to secondary school. The educational distribution of participants cuts across the population in the two counties, again representing the variability among the population in the region. Two parents practicing traditional religious practices had no formal education. The socio-economic status (SES) of the participants was not ascertained, although in these communities, educational level is associated with SES [15]. The professionals included special needs teachers, clinicians, and social workers. Professional groups utilized in this study were of those groups in regular contact with parents of children with ASD. Participants from the two counties did not know each other and those interviewed were different from those in the focus group discussion (FGD).

### Sample size

A total of 103 participants took part in this study, 60 from Kilifi County and 43 from Mombasa County. Table 1 gives details.

**Table 1:** Demographic details of participants.

Group	Educational level	Religious affiliation	Age Range	Participants: Kilifi County	Participants: Mombasa County	Total
Parents of children with autism	0-Secondary school	Traditional, Christianity, Islam	25–75 years	30	21	**51**
Special needs teachers	Certificate-first degree	Christianity, Islam	30–52 years	16	12	**28**
Clinicians	Diploma-first degree	Christianity, Islam	35–48 years	12	8	**20**
Social Workers	Diploma	Christianity	35–50 years	2	2	**4**
**Total**				**60**	**43**	**103**

### Sampling procedures

A purposive-convenience sampling procedure was used to recruit the study participants. This procedure was considered appropriate for several reasons. First, we were targeting families of children with a diagnosis of ASD and professionals who were working with children who had a diagnosis of ASD. Given that this is uncommonly recognized condition and the diagnosis is not routinely made, a random selection would not have provided us with the required sample size. Parents of children with ASD from Kilifi were recruited from the neuro-assessment clinic at the Kilifi District Hospital (KDH). At the KDH, diagnosis of the children was done by a clinician with a background in pediatric neurology in consultation with special education teachers. Participants from Mombasa were selected through the Educational Assessment and Resource Centre (EARC). The EARC team comprises two assessment teachers, three hospital rehabilitators (a physiotherapist, an occupational therapist, and an orthopedic technician), a pediatrician, an ear-nose-throat specialist, and a social worker. An evaluation report from the team is necessary before a child is put into a special education program. Since there is no validated screening and diagnostic measure for ASD in Kenya, presumptive diagnosis was used for sampling children with ASD from the clinical and assessment centers. It is quite likely that different methods of ASD diagnosis had been used in these centers to assess the children. Special needs teachers were recruited from special units and schools, while clinicians were from the county hospitals. Social workers were selected from the Ministry of Culture and Social Services.

### Data collection methods

In-depth interviews and FGD were the data collection methods. Thirty-seven participants from both Kilifi and Mombasa participated in the in-depth interviews that lasted for thirty minutes each, while 66 participants from the two counties took part in the FGD, which each lasted for an hour. The interviews and focus groups were facilitated by the first author (JKG). An interview schedule guided by questions sampled from the explanatory model [12] was used. ASD was described to parents in relation to difficulty in communication with repetitive and restricted observable behaviors due to lack of a local name for autism in this region. Below are the sampled questions.

To parents:

1. What do you think caused your child’s condition?
2. What do you think the condition does to your child?
3. What kind of treatment do you think should be given to your child?
4. What important results do you hope your child to get from the treatment?

To professionals:

1. What do you and parents think are possible causes of autism?
2. What treatment options do parents seek for their child with autism?
3. What expectations do parents have on the treatment given?

### Data storage and analysis

The interviews and the FGD were recorded, transcribed translated into English, and imported to the NVIVO 7 programme (http://www.qsrinternational.com/products_previous-products_nvivo7.aspx) for storage and management. Content analysis as described by Taylor-Powel and Renner [16] was utilized to analyze the data. The text was read thrice for familiarization to identify key issues. The data were coded using free nodes to identify consistencies and differences. All the free nodes with similar messages were grouped into tree nodes each bearing a name of a theme. Connections within and between themes were identified for interpretation. One of the co-authors (AA) went through the transcripts independently for inter-rater triangulation. Conflicting ideas were jointly discussed and analysis was utilized to clarify interpretations for consensus.

### Ethical Approval

Through written informed consents, participants were told that participating in the study was voluntary and there were no subsequent consequences for refusal or withdrawal. The study was approved by the Scientific Steering Committee of Kenya Medical Research Institute in Nairobi, Kenya (SSC #2270). The ethical issues including the approval of the informed consent forms were done by the National Ethics and Review Committee of the Kenya Medical Research Institute based in Nairobi, Kenya. An informed written and signed consent form was obtained from all the study participants.

## Results

**Fig 1:**
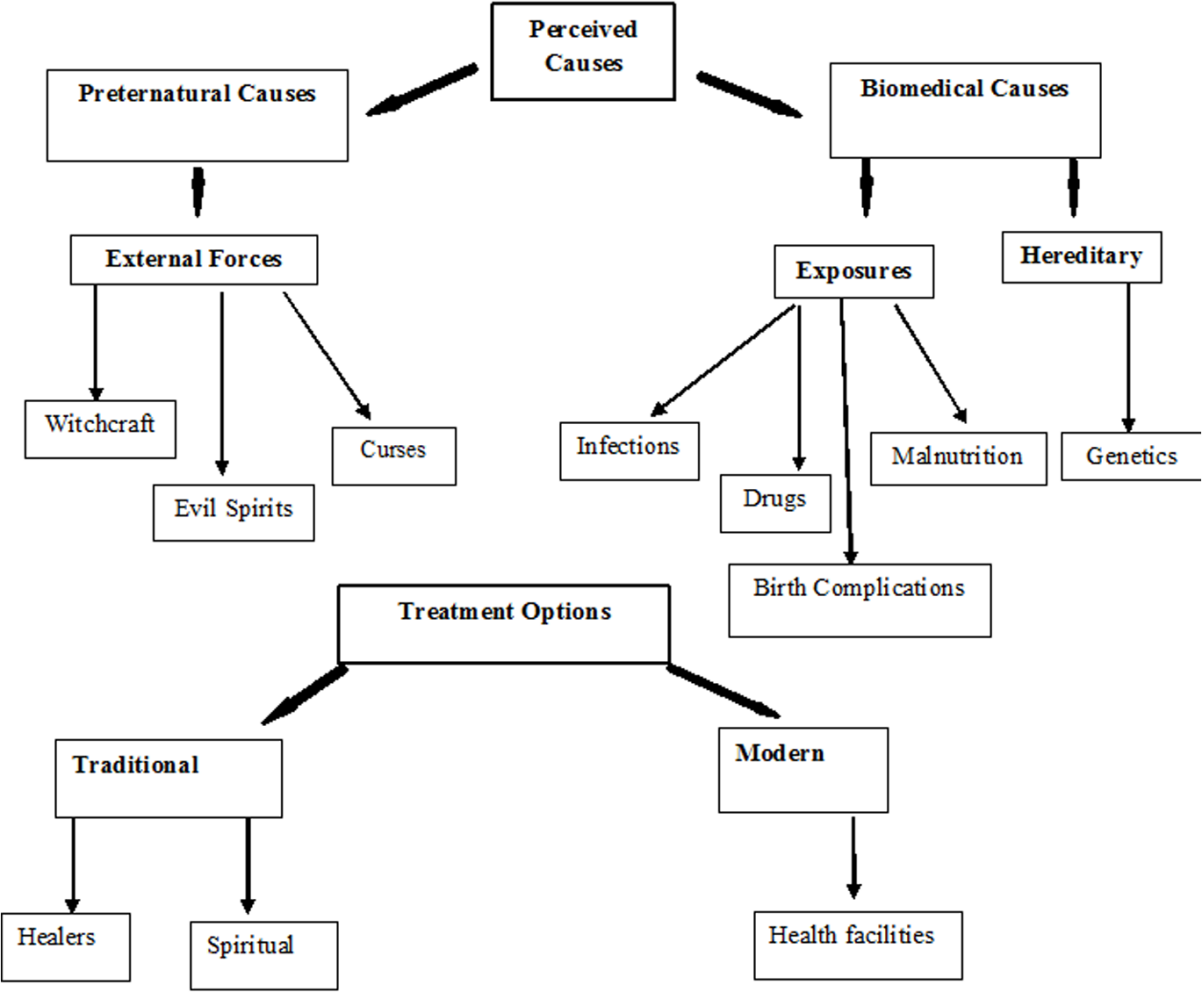
Schematic diagram of themes. Bold arrows point to main themes and sub-themes generated and thin arrows indicate the outcomes from the themes and sub-themes.

These findings present the views of parents and professionals from diverse-cultural settings on perceived causes and treatment options of ASD. Despite cSultural differences, participants expressed opinions with important similarities. Perceived causes of ASD were of two types: Preternatural and biomedical causes. Preternatural causes originated from external forces; the biomedical causes referred to two aspects: exposure and hereditary causes. Treatment options were of two types: traditional treatment and modern treatment, while treatment expectations were in the form of hope for a cure. Fig 1 gives a detailed thematic diagram and S1 File gives quotes generated from study participants.

### Preternatural causes

Preternatural causes that involved external forces happening included witchcraft, evil spirits and curses. Majority of the participants believed that evil spirits could invade a child’s mind and take control of their whole being. This scenario could happen before birth or early childhood.

*“They said that when I was pregnant I passed through a place and I was possessed by evil spirits and that affected my child”* [FGD, parent from Kilifi]

*“Sometimes you think maybe it is evil spirits that have been sent to possess him*.*”* [Interview, parent from Mombasa]

Evil spirits locally called ‘jinnies’ were also believed to cause ASD in this region. In this context, a ‘jinni’ is an evil spirit believed to be kept by an individual to bring wealth or evil happenings to others.

*“…bought a jinni and was keeping*, *or the jinni sitting on the child*, *something of the sort*.*”* [FGD, teacher from Kilifi]

*“Even at the place where I live they say that I have jinnies*. *I have put the child in custody so that I can get money*.*”* [Interview, parent from Kilifi]

Witchcraft was another external force perceived by majority of participants from both settings as a possible cause of ASD. In their opinion, ASD was considered to be a direct manifestation of sorcery.

*“I was seeking for opinions from the people and they are telling me that the child had been bewitched*.*”* [Interview, parent from Kilifi]

*“Most of the times many people think maybe it were because of witchcraft*.*”* [Interview, parent from Mombasa]

*“You know Mr X*, *they associate it with things of witchcraft and curses and things like that*.*”* [FGD, teacher from Mombasa]

*“I think parents believe it is witchcraft*.*”* [Interview, social worker from Kilifi]

Participants from both counties perceived ASD as resulting from a curse parents received after having transgressed taboos or having committed sins against God. Example of wrong-doing that could result in having a child with ASD was infidelity or marrying a relative. Marrying a close relative was seen as against the culturally acceptable norms, especially in Kilifi.

*“I was surprised because everyone was saying that it was ‘kirwa (a curse)*.*’ That one of his parents slept with another person during pregnancy*. *But they had not done so*.*”* [Interview, social worker from Kilifi]

*“I was told that it was either the father or I who did it (had an extramarital affair) or it is called ‘kirwa’*.*”* [FGD, parent from Kilifi]

*“This aspect I did not find it only in Kilifi but also here in Mombasa people relate it to ‘kirwa’*. *This is when a husband has another relationship outside wedlock*.*”* [Interview, clinician from Mombasa]

*“Maybe for example somebody can break the rules of the community*, *so God can punish through that*.*”* [Interview, teacher from Mombasa]

### Biomedical causes

On the aspect of biomedical causes, exposures and hereditary causes were the main issues raised by participants. Exposures to infections, and drugs were perceived to cause ASD. Infections leading to brain damage were highlighted.

*“Maybe we say malaria because when it is cerebral malaria it triggers the brain of the child*, *and the child starts fitting and from that the brain is not the same again and is different from the others*.*”* [Interview, parent from Kilifi]

*“I am also surprised*. *According to me I think in both pregnancies I had malaria complications and also I think it is from the complications and the medication I took*, *that is what caused*.*”* [Interview, parent from Mombasa]

*“…other things like malaria can cause autism*.*”* [Interview, clinician from Mombasa]

*“My views are that it is a brain disorder but it can be precipitated by a child having severe malaria that can affect the brain*.*”* [Interview, teacher from Mombasa]

Perceived inappropriate use of drugs during pregnancy was mentioned as a possible cause of ASD. This aspect was noted by parents from Mombasa and professionals. According to these participants, malaria drugs taken during pregnancy and family planning pills were perceived as a possible danger to the growing fetus.

*“According to me I took malaria drugs when I was pregnant*. *So I think it is from the drugs I took that caused this problem*.*”* [FGD, parent from Mombasa]

“*Some parents think that some family planning methods (pills) cause autism*.*”* [Interview, social worker from Kilifi]

Harmful habits such as alcohol use and smoking by pregnant mothers were perceived to cause ASD by professionals from both counties.

*“You know some mothers*, *they take alcohol during the first three months of pregnancy*, *and they smoke cigarettes*, *all these things will be directly transmitted to the fetus in the womb and this can affect the child’s to get such a disease*.*”* [FGD, teacher from Kilifi]

Malnutrition and birth complications were also perceived to cause ASD. Parents of children with ASD, clinicians and special needs teachers regarded malnutrition as a possible cause. They believed that this was for two reasons: lack of essential nutrients when a mother is pregnant; and a child not receiving regular nutritious meals.

*“Poor balanced diet during pregnancy can be one of the causes*.*”* [Interview, parent from Mombasa]

*“Sincerely I have not investigated or asked but I think it is lack of certain minerals or nutrients that the child is lacking*.*”* [Interview, teacher from Kilifi]

Complications during birth in the form of prolonged labor and use of force were also perceived as possible causes of ASD by most participants.

“*On my side I can say that when I gave birth to my child she did not cry and I had prolonged labor and nurses forced me to push*.” [FGD, parent from Kilifi]

*“When I gave birth to her she had her head squeezed*. *When I was pushing she squeezed the head and was injured and did not cry*.*”* [Interview, parent from Mombasa]

On the hereditary element, genes were viewed to play a role on causing ASD in children. Parents and professionals thought ASD could be passed from parents to the unborn child.

*“You know these sometimes we inherit them from our old guys*. *Maybe in our family there was somebody who was suffering from such a disease and I am very sure*, *it can be a grandchild*, *one of them must have this disease*. *We have seen these things happening*, *and you are told “Your grandfather was suffering from such a disease” so it happens to one of the members of the family*.*”* [Interview, teacher from Mombasa]

*“I’m told it can be passed from my blood to the child’s blood*. *It can be passed in families*.*”* [FGD, parent from Kilifi]

### Treatment options

Treatment options mentioned were of two forms: traditional treatment and modern treatment. Traditional treatment highlighted by most participants involved consultations with traditional healers and spiritual prayers offered to God.

*“I went to a ‘mganga’ (healer)*. *He gives ‘vuo’ (leaves mixed with water) to take for 7 days and liquid medication to apply over his body*. *When it failed to work*, *I looked for help from hospital*.*”* [FGD, parent from Kilifi]

*“I took my child to a traditional healer because I heard that he helped someone*. *He was given medication and fumigation*.*”* [FGD, parent from Mombasa]

*“I go for prayers and the man of God told me that the child has no problem*. *It is God who has blessed me with a child like that*. *He will pray for him and God will perform a miracle*.*”* [Interview, parent from Mombasa]

*“She went with them*, *and when they came back the children explained that aunt took them to a pastor for prayers for the evil spirit to leave*. *So I go for spiritual healing*. [FGD, parent from Kilifi]

The modern treatment that was suggested by most of the participants was the use of health facilities that were available in the counties.

*“We took her to hospital and were referred for therapy and after the therapy her behaviors showed some improvements*.*”* [FGD, parent from Kilifi]

*“Yes*, *sometimes we carry the burden of taking the child to hospital*, *and then we give up*. *Then you later come and regret and start again going for therapy*. *It is good*.*”* [FGD, parent from Mombasa]

*“I only sought treatment from hospital and the physiotherapy exercises helped*.*”* [Interview, parent from Mombasa]

*“It took a long time to attain that (desired behaviors) but the exercises (mazoezi) worked*.*”* [FGD, parents from Kilifi]

*“We continued with therapy on particular dates and we used to come in all appointments for the exercises*. *We are thankful”* [FGD, parents from Mombasa]

### Treatment expectations

Hope for a cure from the treatments given was the main expectation from the parents as ASD was associated with disasters or bad omen to the families and the wider community.

*“We would be grateful if doctors would find medication so that we can have hope of these children would come to heal but we do not know what medication will heal these children*.” [Interview, parent from Mombasa]

*“When I was still in school there was one boy who was like that and during the rainy season everyone would isolate him due to lightning and thunder*. *During that period it is believed that if you stand close to this child you will be struck by the lightning*. *So*, *a cure was needed to avert calamities*. [Interview, social worker from Kilifi]

*“Okay according to my community*, *they believe that these kinds of people are just like a curse from God*. *This is why parents badly need a cure for autism*.*”* [FGD, teacher from Mombasa]

*“I was confused*, *and was told that this child is not talking so I was to take him to the traditional healer and have his “tongue “cut*. *I took him and his “tongue” was cut but no improvement*.*”* [Interview, parent from Kilifi]

## Discussion

This study investigated perceived causes of ASD on the Kenyan Coast with key community informants from diverse cultural backgrounds and religious persuasions. Preternatural causes that involved the manifestation of evil spirits, witchcraft, and curses were major issues of concern mentioned in both settings. Biomedical causes that included exposures to harmful organisms and genetic elements were also viewed as possible causes of ASD. Treatment options that seemed to be mostly preferred for the treatment of ASD in children were those that involved traditional healers, prayers, and taking children to hospital.

These findings suggest that cultural beliefs about evil spirits, witchcraft, and curses cut across the population and religious divide in this part of Kenya. Considering the socio-economic disparity between the two counties [14], the results further suggest that the socioeconomic status of people in this region is less relevant than the impact cultural beliefs and traditional practices have on the people. Cultural values and accepted norms have been seen to influence peoples’ cut-off point of normal variability to that of actual disorder, like ASD [17].

Some disability studies in Africa have further associated witchcraft, evil spirits and curses with causes of other disabilities [18–20], emphasizing the conceptualization of culture in the African context. The results of this study reveal that within this culture, there are traditions and customs that ought to be observed. Failure to observe these traditions could have repercussions possibly leading to a curse that could result into ASD. Studies conducted in Kenya have shown that preferences are given to traditional healers in treatment of epilepsy and disability because these conditions are believed to be caused by evil powers [21,22]. Traditional healers are believed to be well placed in treating calamities associated with cultural beliefs and practices.

Participants also highlighted biomedical causes of ASD in the form of brain insult, malnutrition, misuse of drugs, and perinatal complications. The mention of biomedical causes by participants from both counties demonstrates that cultural and biomedical beliefs are not mutually exclusive on the Kenyan Coast. The perceived relevance of biomedical aspects could indicate culture change in this part of Kenya, mostly in urban settings. Contemporary African culture combines the old and new, a mixture of traditional beliefs with diverse stages of globalization [23]. The diverse opinions of the participants on perceived causes of ASD suggest that the community was beginning to view ASD as a disorder that could have cultural and medical implications.

The results indicate that parents from various cultural backgrounds attend to both traditional and biomedical treatment for their child with ASD in these counties. Traditional and spiritual healers were consulted by parents in pursuit for medications that could cure ASD. These consultations were conducted simultaneously with medical consultations, but sometimes they were carried out after visiting a number of health facilities without success. Studies have shown that healthcare providers often have poor understanding of the symptoms, prognosis and treatment of ASD [24,25]. This could lead to underestimation of the parents’ distress and need for information. Due to this knowledge gap by the health professionals, treatment decisions taken by parents seemed to be influenced by parents’ beliefs on the cause of the child’s ASD.

Beliefs in witchcraft, evil spirits and curses have negative consequences on the child and their parents, hence the need for cure. Due to the negative connotations associated with autism within the community, the results suggest that parents would have a great relief if their child could be cured of the ASD disorder through the treatments they embrace. We have found similar findings for other disabilities on the coast region of Kenya [22]. This emphasizes the desperation of parents in their search for a cure for their child and to avert community gossips and isolation.

However, there is substantial evidence from the results to suggest that there were positive effects from the hospital based treatment. Children with autism who are seen in hospitals exhibit mostly communication problems, mild ataxia and repetitive behaviors. There are no psychologists or speech and language therapists. Thus these children are referred to the departments of occupational and physiotherapy and most parents found physiotherapy as a positive experience.

### Limitations

This study utilized 103 participants from two out of six counties of the Kenyan Coast region. Therefore the findings may not be applicable to people of excluded counties or other counties in Kenya. We did not establish the socio-economic status of the participants which could have increased the validity of the information collected. The participants were not asked about the type of treatment they expected to receive for their child, although most parents were seeking a cure. This would have given us the scope of treatment options they expected from the health facilities. Another limitation of this study is the limited number of participants without formal education who participated. This sample bias may have influenced our results. Given that our sampling frame was broad, it is unlikely that a more representative sample would have identified different themes, such as a difference in etiological beliefs or treatment options. However, education-related variables, such as the relative proportion of parent’s beliefs in preternatural causes and biomedical causes were different in our sample than in the general population.

### Implications

Cultural values influence diagnosis, treatment and the welfare of families with an ASD child [26]. In keeping with studies on epilepsy, and other conditions, it seems like the populations at the Kenyan coast maintain pluralistic beliefs of autism with supernatural and biomedical causes. These results have important implications for setting up treatment and intervention services for children with ASD in our context and other similar contexts. A successful program needs to be sensitive to cultural nuances in terms of beliefs on causation and treatment seeking patterns. Health and social care practitioners must explore families’ beliefs and the impact of their beliefs, because health information that resonates with parents’ beliefs is more likely to be accepted and lead to changes in behavior than those which contradict these beliefs [27]. Attempts to challenge or draw parents away from their systems of understanding can cause confusion and breakdown in communication.

## Conclusions

Findings from this study demonstrate that people from diverse cultural backgrounds on the Kenyan Coast have similar beliefs around perceived causes of ASD. The insights gained from this study can be used to develop intervention programmes aimed at creating awareness on ASD, and encouraging optimal health seeking behavior among parents of children with ASD and other neurodevelopmental disorders.

## Supporting Information

S1 File Gives quotes relating to different themes generated from the study participants. (DOC)
