# Supplementary material for: Parents’ and Professionals’ Perceptions on Causes and Treatment Options for Autism Spectrum Disorders (ASD) in a Multicultural Context on the Kenyan Coast
Source: PLoS One. 2015 Aug 12;10(8):e0132729. doi: 10.1371/journal.pone.0132729 (PMC4534101; doi:10.1371/journal.pone.0132729)
Supplement: S1 File — (DOC) [file pone.0132729.s001.doc]

**S1. Supporting Information**

**Ms. No: PONE-D-14-55529: Parents’ and Professionals’ Perceptions on Causes and Treatment Options for Autism Spectrum Disorders (ASD) in a Multicultural Context on the Kenyan Coast**

**Causes**

**Evil spirits**

[<Internals\\FGDs\\CONVERSATION BETWEEN KEN AND SPECIAL SCHOOL TEACHERS >](../b2bd4a91-1a70-4f4c-81d0-66424faf957b) - § 2 references coded [2.40% Coverage]

Reference 1 - 1.43% Coverage

Then another cause may be when i got the baby the passer-by noticed the child had a problem, and according to them we went and bought a jinni from Swahili people and the jinni was keeping or sitting on the child something of the sort

Reference 2 - 0.97% Coverage

Now it was like they were also monitoring the child. One time my husband got some scholarship and he went, now they concluded, “it is true they have a jinni”

[<Internals\\FGDs\\FGD TRANSLATION WITH PARENTS ASD IN MOMBASA>](../2480ad09-0f47-4b70-99d0-61a1cc35564c) - § 2 references coded [7.47% Coverage]

Reference 1 - 1.72% Coverage

Some think maybe the child has been offered for sacrifice for that the parent can get wealth in return.

Reference 2 - 5.75% Coverage

They say it is me who has done that to get rich or my people to get rich. Another thing they say is that when I was pregnant I passed through a place where someone had put evil spells. So when I passed though that place, that evil got into me. But that evil was not meant for me, but because of my bad luck I got into it and it went into my stomach.

[<Internals\\FGDs\\FOCUS GROUP DISCUSSION WITH MOTHERS OF ASD CHILDREN>](../241aa87c-88d3-4933-a8cf-41cdf190c924) - § 2 references coded [2.96% Coverage]

Reference 1 - 2.22% Coverage

It is being said because other children when seen to be that way they say that it is not caused by malaria but it is just because one is keeping jins so malaria is just an excuse. At that time you might be sleeping without food and you are always on the run day and night looking for what will sustain you but they will tell you that you are keeping jins so you are doing that so that you can be rich.

Reference 2 - 0.75% Coverage

When you give birth to such a child and you are rich, they say “you have make him seat of jinnie” and the richness is there because of the child.

[<Internals\\FGDs\\FOCUS GROUP DISCUSSION WITH PARENTS WHO HAVE CHILDREN WITH AUTISM 1>](../86ece053-4338-442d-afcf-2c0150841291) - § 6 references coded [2.80% Coverage]

Reference 1 - 0.26% Coverage

They also said that when I was pregnant I passed through a place and was possessed by evil spirit and that affected the child.

Reference 2 - 0.28% Coverage

They said that he had been possessed by evil spirit because when I was pregnant I was taken to the old buildings in Mombasa.

Reference 3 - 0.34% Coverage

My husband said may be; because I saw a cat there and it scared me, so he said may be this happened because of the day that I was scared by the cat while I was pregnant.

Reference 4 - 0.71% Coverage

Yes I saw a cat and was questioning the quietness of the place; and I saw the cat and he said that the cat can go and report and say that it has seen a person and I was scared. It stayed in his mind (husband) and when I gave birth and the child started the problem he related it to that day we saw the cat.

Reference 5 - 0.55% Coverage

They were treating Kirwa , mapepo, and they were telling me that I have an evil spirit that doesn’t love children but when I see it the children I gave birth before him are okay and they are saying that I have a pepo who doesn’t love a baby boy while my first born is even a boy

Reference 6 - 0.66% Coverage

When you go with her out of the home people become surprised because of her actions. Some say that she was thrown a jinni but you do not know because we are born again. And some say that we did some mistakes in the church and we need to repent for her to walk and talk.

[<Internals\\Interviews\\Social worker Malindi>](../84f6410a-212e-4976-a6cf-49acd2588473) - § 1 reference coded [5.50% Coverage]

Reference 1 - 5.50% Coverage

Those people with these children tend to hide them, they don’t want to expose them to the society because they are afraid people are going to say what is wrong with their child, and maybe the society is going to say the child is possessed by demon

[<Internals\\Interviews\\CONVERSATION BETWEEN GONA AND MAMA FELIX>](../4580088f-1a38-4c40-a9cf-2408dff57b1a) - § 1 reference coded [1.79% Coverage]

Reference 1 - 1.79% Coverage

Even at the place where I live, they all say that I have jins; I have put the child in custody of the jins so that I get money; I have built a house because of that child.

[<Internals\\Interviews\\CONVERSATION BETWEEN GONA AND MAMA TEDDY>](../39a7b590-b1ad-4cb9-98cf-20ccc4c0c973) - § 2 references coded [2.09% Coverage]

Reference 1 - 0.56% Coverage

Sometimes you think maybe it is evil spirits that have been thrown to him.

Reference 2 - 1.52% Coverage

There were people who were telling me that this child was good and maybe there was a person who was jealousy and threw evil spirits to him. When you are desperate, you believe in everything you are told.

[<Internals\\Interviews\\INDEPTH INTERVIEW WITH A PARENT (2)>](../eff41e1a-749c-45d0-b0d0-66426de7d63d) - § 1 reference coded [4.58% Coverage]

Reference 1 - 4.58% Coverage

Some people here in Mombasa might be saying that we are using the child to get wealth.

§ 2 references coded [2.40% Coverage]

Reference 1 - 1.43% Coverage

Then another cause may be when Igot the baby the passer-by noticed the child had a problem, and according to them we went and bought a jinni from Swahili people and the jinni was keeping or sitting on the child something of the sort

Reference 2 - 0.97% Coverage

Now it was like they were also monitoring the child. One time my husband got some scholarship and he went, now they concluded, “it is true they have a jinni”

[<Internals\\FGDs\\FGD TRANSLATION WITH PARENTS ASD IN MOMBASA>](../2480ad09-0f47-4b70-99d0-61a1cc35564c) - § 2 references coded [7.47% Coverage]

Reference 1 - 1.72% Coverage

Some think maybe the child has been offered for sacrifice for that the parent can get wealth in return.

Reference 2 - 5.75% Coverage

They say it is me who has done that to get rich or my people to get rich. Another thing they say is that when I was pregnant I passed through a place where someone had put evil spells. So when I passed though that place, that evil got into me. But that evil was not meant for me, but because of my bad luck I got into it and it went into my stomach.

[<Internals\\FGDs\\FOCUS GROUP DISCUSSION WITH PARENTS WHO HAVE CHILDREN WITH AUTISM 1>](../86ece053-4338-442d-afcf-2c0150841291) - § 6 references coded [2.80% Coverage]

Reference 1 - 0.26% Coverage

They also said that when I was pregnant I passed through a place and was possessed by evil spirit and that affected the child.

Reference 2 - 0.28% Coverage

They said that he had been possessed by evil spirit because when I was pregnant I was taken to the old buildings in Mombasa.

Reference 3 - 0.34% Coverage

My husband said may be; because I saw a cat there and it scared me, so he said may be this happened because of the day that I was scared by the cat while I was pregnant.

[<Internals\\Interviews\\Social worker Malindi>](../84f6410a-212e-4976-a6cf-49acd2588473) - § 1 reference coded [5.50% Coverage]

Reference 1 - 5.50% Coverage

Those people with these children tend to hide them, they don’t want to expose them to the society because they are afraid people are going to say what is wrong with their child, and maybe the society is going to say the child is possessed by demon

[<Internals\\Interviews\\CONVERSATION BETWEEN GONA AND MAMA TEDDY>](../39a7b590-b1ad-4cb9-98cf-20ccc4c0c973) - § 2 references coded [2.09% Coverage]

Reference 1 - 0.56% Coverage

Sometimes you think maybe it is evil spirits that have been sent to possess him.

Reference 2 - 1.52% Coverage

There were people who were telling me that this child was good and maybe there was a person who was jealousy and threw evil spirits to him. When you are desperate, you believe in everything you are told.

**Witchcraft**

[<Internals\\FGDs\\FGD TRANSLATION WITH PARENTS ASD IN MOMBASA>](../2480ad09-0f47-4b70-99d0-61a1cc35564c) - § 2 references coded [5.72% Coverage]

Reference 1 - 3.00% Coverage

Others say maybe on the side of the mother or father someone was a witchdoctor, and s/he died nobody took over, that’s why it was now turned to the child and it is disturbing him.

Reference 2 - 2.72% Coverage

In our family we are all saved, but neighbors think maybe someone is behind that, maybe he was given something to eat which affected the child, things like that.

[<Internals\\Interviews\\Social worker >](../84f6410a-212e-4976-a6cf-49acd2588473) - § 1 reference coded [7.73% Coverage]

Reference 1 - 7.73% Coverage

They don’t take it normally, though the cases are not common but from my observation most of them believe it as witchcraft oriented, they don’t believe it’s a medical condition that need medical attention that can be attended to, the child can be helped out. What they believe they go to a witch doctor to be attended to that what they believe.

[<Internals\\Interviews\\CONVERSATION BETWEEN GONA AND MAMA FELIX>](../4580088f-1a38-4c40-a9cf-2408dff57b1a) - § 2 references coded [1.93% Coverage]

Reference 1 - 0.60% Coverage

When I tell someone that way she/he says “This child must have been bewitched”.

Reference 2 - 1.32% Coverage

I was asking/seeking for opinions from people and they were telling me that the child had been bewitched.

[<Internals\\Interviews\\CONVERSATION BETWEEN GONA AND MAMA LEAH>](../873e28ae-c4d6-47c7-accf-24090ce1ad71) - § 2 references coded [2.62% Coverage]

Reference 1 - 0.63% Coverage

Most of the times most people think that maybe it is because of witchcraft

Reference 2 - 1.99% Coverage

In Swahili they believe that if one wants to be rich he/she has to sacrifice a child to be rich and something like that. They take it that you have kept that child in that condition so that you can be rich but I don’t believe in that.

So they believe that one may be was bewitched. You know bewitching amongst our community is very common so whatever happens, they just say “this one was bewitched”

Reference 2 - 1.27% Coverage

That is why when a child is born while having that problem of autism, he as a parent they will first go to the witchdoctors, and find solutions from there believing that the child might have been bewitched.

[<Internals\\FGDs\\FGD TRANSLATION WITH PARENTS ASD IN MOMBASA>](../2480ad09-0f47-4b70-99d0-61a1cc35564c) - § 2 references coded [5.72% Coverage]

Reference 1 - 3.00% Coverage

Others say maybe on the side of the mother or father someone was a witchdoctor, and s/he died nobody took over, that’s why it was now turned to the child and it is disturbing him.

Reference 2 - 2.72% Coverage

In our family we are all saved, but neighbors think maybe someone is behind that, maybe he was given something to eat which affected the child, things like that.

[<Internals\\Interviews\\Community Nurse Malindi>](../84f6410a-212e-4976-a6cf-49acd2588473) - § 1 reference coded [7.73% Coverage]

Reference 1 - 7.73% Coverage

They don’t take it normally, though the cases are not common but from my observation most of them believe it as witchcraft oriented, they don’t believe it’s a medical condition that need medical attention that can be attended to, the child can be helped out. What they believe they go to a witch doctor to be attended to that what they believe.

[<Internals\\Interviews\\CONVERSATION BETWEEN GONA AND MAMA FELIX>](../4580088f-1a38-4c40-a9cf-2408dff57b1a) - § 2 references coded [1.93% Coverage]

Reference 1 - 0.60% Coverage

When I tell someone that way she/he says”This child must have been bewitched”.

Reference 2 - 1.32% Coverage

Now I was asking/seeking for opinions from people and they were telling me that the child had been bewitched and I was telling them that this child had not been bewitched.

[<Internals\\Interviews\\CONVERSATION BETWEEN KEN AND ASSESSOR KILIFI>](../097a82ea-7c96-41a8-a2cf-49acd29d8c50) - § 1 reference coded [0.44% Coverage]

Reference 1 - 0.44% Coverage

You know them Mr X, they associate it with things of witchcraft and curses and things like that.

[<Internals\\Interviews\\CONVERSATION BETWEEN KEN AND NURSE KACHE>](../2039b087-41ee-4f0b-8acf-49acd3279ccc) - § 1 reference coded [3.87% Coverage]

Reference 1 - 3.87% Coverage

Our community as Mijikenda, believe that these children are bewitched or it is a certain curse from forefathers or ancestors and some blame the hospital maybe one went and gave birth to the hospital and so on.

[<Internals\\Interviews\\CONVERSATION BETWEEN KEN AND SOCIAL WORKERS MOMBASA>](../18e2ec4b-44ae-4bcc-a9cf-49acd35e5bb4) - § 1 reference coded [0.49% Coverage]

Reference 1 - 0.49% Coverage

Some I think that they are bewitched.

[<Internals\\Interviews\\INTERVIEW WITH A MEDICAL DOCTOR IN MOMBASA>](../badc1b3b-421d-40ff-b9d0-61a1dffc3343) - § 1 reference coded [1.48% Coverage]

Reference 1 - 1.48% Coverage

They are those who might think it is witchcraft.

[<Internals\\Interviews\\INTERVIEW WITH PARENT IN MOMBASA>](../d1db6edc-c8b1-4e31-b7d0-61a1e02259d8) - § 2 references coded [14.78% Coverage]

Reference 1 - 8.61% Coverage

One is that they think it is witchcraft. It depends on where one comes from. Ok…the people I have dealt before say it is witchcraft from this group or from this group; or the child was bewitched or someone looked at the boy with bad eyes

Reference 2 - 6.17% Coverage

Sometimes they say it is bad talk from the neighborhood; the boy was developing well and everyone admired the boy; now he is like that. Everything goes to the parents.

**Curses**

[<Internals\\FGDs\\CONVERSATION BETWEEN KEN AND SPECIAL SCHOOL TEACHERS >](../b2bd4a91-1a70-4f4c-81d0-66424faf957b) - § 11 references coded [10.21% Coverage]

Reference 1 - 0.62% Coverage

Okay according to my community, they believe that these kinds of people is just like curse from God.

Reference 2 - 0.62% Coverage

Maybe for example somebody can break the rules of their community, so God can punish you through that

Reference 3 - 0.67% Coverage

Maybe marrying your cousin, somebody you relate to him or her. The impact may be having a child with autism.

Reference 4 - 1.56% Coverage

Another one is there is a curse, may be the grand grandparents did something wrong so that thing follows the line. So that one is a belief that something is wrong or one of the grandparents made something wrong so the rest of the generation is suffering.

Reference 5 - 1.05% Coverage

Another thing is that, in my community, they think that may be these parents may be they relate so as a result of that, then the child is born with that problem of autism.

Reference 6 - 1.02% Coverage

One as they have said is the mixture of blood, which when you marry people of the same blood for example an uncle marrying sister’s daughter, that one can cause autism

Reference 7 - 1.09% Coverage

This curse might come may be a parent had done something from the great grandfathers, and if it was not cleansed it will affect the children within the family, so this is a curse

Reference 8 - 1.16% Coverage

Another one is if a woman is married and the dowry is not paid in terms of either a cow, blanket or the alcohol it should be paid. If it is not paid, a disability will happen in your family.

Reference 9 - 1.32% Coverage

According to me with the experience I passed through, okay I can say my husband is my cousin, so may be mixing the blood , that blood which has been mixed i think caused the problem and made that child to be hyper.

Reference 10 - 0.28% Coverage

So mixing of blood when people are so close.

Reference 11 - 0.82% Coverage

According to my view towards autism, is that autism can come as a result of mixing blood, which is marrying somebody whom you relate.

[<Internals\\FGDs\\FGD TRANSLATION WITH PARENTS ASD IN MOMBASA>](../2480ad09-0f47-4b70-99d0-61a1cc35564c) - § 1 reference coded [1.75% Coverage]

Reference 1 - 1.75% Coverage

I think maybe it was because I had stayed for a very long time without having gone to say hi to my parents.

[<Internals\\FGDs\\FOCUS GROUP DISCUSSION WITH MOTHERS OF ASD CHILDREN>](../241aa87c-88d3-4933-a8cf-41cdf190c924) - § 2 references coded [0.74% Coverage]

Reference 1 - 0.50% Coverage

Is told that that you slept with another man apart from your husband/wife

Reference 2 - 0.23% Coverage

It is normally said that it is the wife.

[<Internals\\FGDs\\FOCUS GROUP DISCUSSION WITH PARENTS WHO HAVE CHILDREN WITH AUTISM 1>](../86ece053-4338-442d-afcf-2c0150841291) - § 4 references coded [1.59% Coverage]

Reference 1 - 0.28% Coverage

When the body was becoming smaller i was told that may be I had slept with another man when I was pregnant as these are traditional things.

Reference 2 - 0.29% Coverage

I was told while she was still young. I was told that it was either me or her dad who did it and it is called “kirwa”, but he body did not change.

Reference 3 - 0.28% Coverage

I was now stark because everyone was saying that it was “kirwa” and I took that step and when I saw that it did not work, I left everything.

Reference 4 - 0.74% Coverage

Sometimes as a human being I feel like someone was unfair to me, or may be when we courted with the husband there might have been something wrong we did. When I reflect back I see that it is not the case because when we courted he was far and I was apart and when he came we were joined in holy matrimony. We have seen where we went wrong but we did not get any answer.

[<Internals\\Interviews\\Community Nurse Malindi>](../84f6410a-212e-4976-a6cf-49acd2588473) - § 1 reference coded [2.50% Coverage]

Reference 1 - 2.50% Coverage

the child of these condition the family is seem be cursed due to something wrong they did to their ancestors.

[<Internals\\Interviews\\INTERVIEW WITH CLINICIAN IN MOMBASA>](../ec35ba3b-1e4d-4a91-bcd0-61a1e0131748) - § 1 reference coded [11.50% Coverage]

Reference 1 - 11.50% Coverage

On the people I have encountered with, many of them mention “chirwa.” This aspect I did not find it only when I was in Kilifi, but also here in Mombasa people relate it to “chirwa”. A good example is the study participant whose mother actually said if it were not for her husband having another relationship outside wedlock when she gave birth to her child, her child would not be like that. So there are those issues where a man has an affair outside his marriage.

[<Internals\\Interviews\\INTERVIEW WITH PARENT ASD IN MOMBASA>](../42110a9d-cd73-44e3-8fd0-61a1e03015b1) - § 1 reference coded [6.00% Coverage]

Reference 1 - 6.00% Coverage

It is a common perception that if a child has a disability then it is suspected that one of the parents or both of them wronged someone or many people who got annoyed and therefore affected the child

**Malaria**

[<Internals\\FGDs\\FGD TRANSLATION WITH PARENTS ASD IN MOMBASA>](../2480ad09-0f47-4b70-99d0-61a1cc35564c) - § 1 reference coded [1.70% Coverage]

Reference 1 - 1.70% Coverage

If a child was admitted in hospital unconscious; maybe the child was sick, then admitted in hospital.

[<Internals\\FGDs\\FOCUS GROUP DISCUSSION WITH MOTHERS OF ASD CHILDREN>](../241aa87c-88d3-4933-a8cf-41cdf190c924) - § 7 references coded [5.93% Coverage]

Reference 1 - 1.02% Coverage

It can be caused by cerebral malaria, and you will see him/her changing or you find out that the brain changes and might even move using the back. I think it can be caused by malaria.

Reference 2 - 1.12% Coverage

May be we say malaria because when it is cerebral malaria it triggers the brain of the child, and the child starts fitting and from that the brain is not the same again and is different from the others.

Reference 3 - 1.12% Coverage

When the child has malaria that has gone to the head, some say it is “nyuni” and go to the traditional healer and get “mavuo” (herbs) and be poured and when it fails then the child is taken to hospital.

Reference 4 - 0.45% Coverage

They will first say that it is nyuni and not malaria, and say that it is nyago.

Reference 5 - 0.52% Coverage

It is malaria that comes through the head. (Cerebral malaria), that is what they call nyago.

Reference 6 - 0.38% Coverage

“Anavungwa”. They take mavuo and chicken then the child anavungwa.

Reference 7 - 1.34% Coverage

First they go to the traditional healers then when he fails we take him/her to hospital and when it succeeds the person will say that the hospital is healing and also when the traditional healer succeeds we say the traditional healer heals.

[<Internals\\Interviews\\INDEPTH INTERVIEW WITH A TEACHER MOMBASA (2)>](../eff41e1a-749c-45d0-b0d0-66426de7d63d) - § 1 reference coded [6.88% Coverage]

Reference 1 - 6.88% Coverage

My views are that it is a brain disorder, but it can be precipitated by a child having severe malaria, that can affect the brain

[<Internals\\Interviews\\INTERVIEW WITH CLINICIAN IN MOMBASA>](../ec35ba3b-1e4d-4a91-bcd0-61a1e0131748) - § 1 reference coded [3.65% Coverage]

Reference 1 - 3.65% Coverage

Ok like (pause) those are probably the commonly seen, but there could be other things that would give you…other things like malaria can give autism.

[<Internals\\FGDs\\CONVERSATION BETWEEN KEN AND SPECIAL SCHOOL TEACHERS AT SAHAJANAND SPECIAL SCHOOL>](../b2bd4a91-1a70-4f4c-81d0-66424faf957b) - § 1 reference coded [0.50% Coverage]

Reference 1 - 0.50% Coverage

Another thing, I think drugs can make woman maybe to deliver child with autism.

[<Internals\\FGDs\\FGD WITH PARENTS OF ASD CHILDREN IN MOMBSA ON 29>](../f86e17d7-c286-4c8d-81d0-61a1cc9a0b78) - § 2 references coded [3.65% Coverage]

Reference 1 - 2.52% Coverage

They say the use of family planning methods like the use of pills could led to that problem.

Reference 2 - 1.14% Coverage

Family planning brings all these problems.

[<Internals\\Interviews\\CONVERSATION BETWEEN GONA AND MADAM FATMA>](../cb9e4191-5e00-404f-8bcf-47501e686507) - § 1 reference coded [1.71% Coverage]

Reference 1 - 1.71% Coverage

but others say it could be because of some of the things which these mothers feed on, which include juices, those which are not fresh juices, the type of food, coke whatever, but yet the causes have not been found.

[<Internals\\Interviews\\CONVERSATION BETWEEN GONA AND MADAM KINGI>](../adb72b59-4036-4175-89cf-47501e7dd104) - § 1 reference coded [3.83% Coverage]

Reference 1 - 3.83% Coverage

When the mother was pregnant, maybe she did not get foods that were well balanced and then there are these harmful pills that we are told not to use when we are pregnant like drugs, alcohol, cigarettes that can affect the child while still in the womb.

[<Internals\\Interviews\\CONVERSATION BETWEEN GONA AND MAMA LEAH>](../873e28ae-c4d6-47c7-accf-24090ce1ad71) - § 2 references coded [1.77% Coverage]

Reference 1 - 1.59% Coverage

I am also surprised. According to me I think in both pregnancies I had malaria complications and also I think it is from the complications and the medication I took, that is what caused.

Reference 2 - 0.18% Coverage

Malaria medication.

[<Internals\\Interviews\\INTERVIEW WITH PARENT IN MOMBASA>](../d1db6edc-c8b1-4e31-b7d0-61a1e02259d8) - § 1 reference coded [2.72% Coverage]

Reference 1 - 2.72% Coverage

there are so many things; or if the mother drinks a bit, all these things.

[<Internals\\Interviews\\INTERVIEW WITH PARENT IN MOMBASA>](../42110a9d-cd73-44e3-8fd0-61a1e03015b1) - § 2 references coded [7.24% Coverage]

Reference 1 - 5.04% Coverage

Yah, we could have some external forces like uses of drugs by the expectant mother. Some drugs can be taken that are dangerous and harmful to the child in the womb.

Reference 2 - 2.20% Coverage

It can be either. It can be the narcotic drug or the prescribed drug.

**Misuse of drugs**

Another thing, I think drugs can make woman maybe to deliver child with autism.

[<Internals\\FGDs\\FGD WITH PARENTS OF ASD CHILDREN IN MOMBSA ON 29>](../f86e17d7-c286-4c8d-81d0-61a1cc9a0b78) - § 2 references coded [3.65% Coverage]

Reference 1 - 2.52% Coverage

They say the use of family planning methods like the use of pills could led to that problem.

Reference 2 - 1.14% Coverage

Family planning brings all these problems.

[<Internals\\Interviews\\CONVERSATION BETWEEN GONA AND MADAM FATMA>](../cb9e4191-5e00-404f-8bcf-47501e686507) - § 1 reference coded [1.71% Coverage]

Reference 1 - 1.71% Coverage

but others say it could be because of some of the things which these mothers feed on, which include juices, those which are not fresh juices, the type of food, coke whatever, but yet the causes have not been found.

[<Internals\\Interviews\\CONVERSATION BETWEEN GONA AND MADAM KINGI>](../adb72b59-4036-4175-89cf-47501e7dd104) - § 1 reference coded [3.83% Coverage]

Reference 1 - 3.83% Coverage

When the mother was pregnant, maybe she did not get foods that were well balanced and then there are these harmful pills that we are told not to use when we are pregnant like drugs, alcohol, cigarettes that can affect the child while still in the womb.

[<Internals\\Interviews\\CONVERSATION BETWEEN GONA AND MAMA LEAH>](../873e28ae-c4d6-47c7-accf-24090ce1ad71) - § 2 references coded [1.77% Coverage]

Reference 1 - 1.59% Coverage

I am also surprised. According to me I think in both pregnancies I had malaria complications and also I think it is from the complications and the medication I took, that is what caused.

Reference 2 - 0.18% Coverage

Malaria drugs.

[<Internals\\Interviews\\INTERVIEW WITH PARENT IN MOMBASA>](../d1db6edc-c8b1-4e31-b7d0-61a1e02259d8) - § 1 reference coded [2.72% Coverage]

Reference 1 - 2.72% Coverage

there are so many things; or if the mother drinks a bit, all these things.

[<Internals\\Interviews\\INTERVIEW WITH PARENT IN MOMBASA>](../42110a9d-cd73-44e3-8fd0-61a1e03015b1) - § 2 references coded [7.24% Coverage]

Reference 1 - 5.04% Coverage

Yah, we could have some external forces like uses of drugs by the expectant mother. Some drugs can be taken that are dangerous and harmful to the child in the womb.

Reference 2 - 2.20% Coverage

It can be either. It can be the narcotic drug or the prescribed drug.

**Drinking and smoking during pregnancy**

[<Internals\\FGDs\\CONVERSATION BETWEEN KEN AND SPECIAL SCHOOL TEACHERS AT SAHAJANAND SPECIAL SCHOOL>](../b2bd4a91-1a70-4f4c-81d0-66424faf957b) - § 1 reference coded [1.78% Coverage]

Reference 1 - 1.78% Coverage

The last one is may be about these drugs and alcohol. You know some mothers, they take alcohol during the first three months of pregnancy, and they smoke cigarettes, all these things will directly transmitted to the fetus in the womb and this can affect the child’s to get such a disease.

[<Internals\\Interviews\\INTERVIEW WITH PARENT IN MOMBASA>](../d1db6edc-c8b1-4e31-b7d0-61a1e02259d8) - § 1 reference coded [6.72% Coverage]

Reference 1 - 6.72% Coverage

What I was thinking about was when I first met the child; I thought the mother was a drunkard; so maybe about the condition I thought that it came about by the mother being a drunkard.

The last one is may be about these drugs and alcohol. You know some mothers, they take alcohol during the first three months of pregnancy, and they smoke cigarettes, all these things will directly transmitted to the foetus in the womb and this can affect the child’s to get such a disease.

[<Internals\\Interviews\\INTERVIEW WITH PARENT IN MOMBASA>](../d1db6edc-c8b1-4e31-b7d0-61a1e02259d8) - § 1 reference coded [6.72% Coverage]

Reference 1 - 6.72% Coverage

What I was thinking about was when I first met the child; I thought the mother was a drunkard; so maybe about the condition I thought that it came about by the mother being a drunkard.

**Complication during birth**

[<Internals\\FGDs\\CONVERSATION BETWEEN KEN AND SPECIAL SCHOOL TEACHERS AT SAHAJANAND SPECIAL SCHOOL>](../b2bd4a91-1a70-4f4c-81d0-66424faf957b) - § 3 references coded [2.57% Coverage]

Reference 1 - 0.17% Coverage

Prolonged labour, accident….

Reference 2 - 1.81% Coverage

Another thing according to the way I experienced, the pains were too severe and it took me shorter time to deliver the baby, the baby was big and the time to deliver the baby was short and the pains were too much, and when I got the baby he cried over one hour, crying as if the child was beaten.

Reference 3 - 0.59% Coverage

maybe the prolonged period labour pains, and also lack of nutrition during the prenatal stage.

[<Internals\\FGDs\\FGD-Teachers special school>](../6d3146cc-0ae0-43b3-bdcf-4d937f81f1b9) - § 1 reference coded [0.30% Coverage]

Reference 1 - 0.30% Coverage

It could be a possible causes during birth the way the babies are produced it is during labor it is a very hard exercise am meant to understand and during this time many things can go wrong and probably this can be the causes of this imbalances.

[<Internals\\FGDs\\FOCUS GROUP DISCUSSION WITH PARENTS WHO HAVE CHILDREN WITH AUTISM 1>](../86ece053-4338-442d-afcf-2c0150841291) - § 2 references coded [0.57% Coverage]

Reference 1 - 0.29% Coverage

When a child is born, s/he normally cries, but for her she did not cry and was born at home because of the problem with transport to the hospital.

Reference 2 - 0.28% Coverage

On my side I can say that, when I gave birth to my child, she did not cry and I had prolonged labor and nurses came and forced me to push.

[<Internals\\Interviews\\CONVERSATION BETWEEN GONA AND MAMA DENIS>](../c06461f5-c612-4d04-83cf-26833fbdb99f) - § 2 references coded [3.43% Coverage]

Reference 1 - 2.48% Coverage

I was explained well during the delivery, they told me that I had prolonged labor so baby had overstayed in the womb, and the umbilical cord had intertwined on the neck, so he could not come out and I had undergone a prolonged labor so when the baby came out, the baby was tired and the brain had been damaged. So, that is what they explained to me.

Reference 2 - 0.94% Coverage

He is the one who explained to me that the child missed oxygen to the brain during delivery, and that is why the brain was damaged.

[<Internals\\Interviews\\CONVERSATION BETWEEN GONA AND MAMA TEDDY>](../39a7b590-b1ad-4cb9-98cf-20ccc4c0c973) - § 1 reference coded [0.77% Coverage]

Reference 1 - 0.77% Coverage

He was just saying may be, or the child was born after a prolonged labor or was born with complications.

[<Internals\\Interviews\\INTERVIEW WITH PARENT IN MOMBASA>](../d1db6edc-c8b1-4e31-b7d0-61a1e02259d8) - § 1 reference coded [4.50% Coverage]

Reference 1 - 4.50% Coverage

She had very prolonged labor. I don’t know if this can be a cause but I was thinking about it. It was very prolonged labor.

**Genetic causes**

[<Internals\\FGDs\\CONVERSATION BETWEEN KEN AND SPECIAL SCHOOL TEACHERS L>](../b2bd4a91-1a70-4f4c-81d0-66424faf957b) - § 2 references coded [3.85% Coverage]

Reference 1 - 1.20% Coverage

Another cause they say that it is a genetic from the mother, from where she comes from. That is why in the olden days before one gets married, the parents were supposed to go to study the family.

Reference 2 - 2.65% Coverage

Another one that I wanted to talk about is genetic. You know these sometimes we inherit them from our old guys. Maybe in our family there was somebody who was suffering from such a disease and I am very sure, it can be a grandchild, one of them must have this disease. We have seen these things happening, and you are told “Your grandfather was suffering from such a disease” so it happens to one of the members of the family.

[<Internals\\FGDs\\FOCUS GROUP DISCUSSION WITH CLINICIANS 1>](../3bb20a44-d114-44ee-becf-475030f7fa58) - § 1 reference coded [0.42% Coverage]

Reference 1 - 0.42% Coverage

It can be environmental thing, some can be due to genetic, and children with congenital chromosomal abnormities can be causes of autistic disorders,

[<Internals\\Interviews\\Community Nurse Malindi>](../84f6410a-212e-4976-a6cf-49acd2588473) - § 1 reference coded [1.56% Coverage]

Reference 1 - 1.56% Coverage

Other cause could be genetic? This is an issue the runs in the family,

[<Internals\\Interviews\\CONVERATION BETWEEN GONA AND MR JONAM>](../4b61e0b3-d110-4eac-accf-47501de57966) - § 1 reference coded [2.95% Coverage]

Reference 1 - 2.95% Coverage

Genetics, some are brought---they come from genes from the previous parents so they are hereditary and I think also that another cause is if child during birth also some form of this mental retardation can come

[<Internals\\Interviews\\CONVERSATION BETWEEN GONA AND MR MUGENDI>](../e7ace36c-36f5-4037-85cf-47501ef4d629) - § 2 references coded [0.92% Coverage]

Reference 1 - 0.28% Coverage

some are conditions which are inherited.

Reference 2 - 0.63% Coverage

From birth some are born like that, maybe it is because of genes may be from the parents,

[<Internals\\Interviews\\CONVERSATION BETWEEN KEN AND SOCIAL WORKERS MOMBASA>](../18e2ec4b-44ae-4bcc-a9cf-49acd35e5bb4) - § 1 reference coded [2.35% Coverage]

Reference 1 - 2.35% Coverage

I can say that may be it is genetically and it was not very common in the past years or people did not know it was autism, may be it is genetic or environmental factors may be.

[<Internals\\Interviews\\CONVERSATION BETWEEN MR>](../f999447e-5110-4dda-a9cf-47501f671999) - § 1 reference coded [2.75% Coverage]

Reference 1 - 2.75% Coverage

Well, I can tend to think that these are conditions that run in family other wise I don’t have any specific cause that I can say this is the cause of autism.

[<Internals\\Interviews\\INDEPTH INTERVIEW WITH A PARENT OF CHILD (2)>](../eff41e1a-749c-45d0-b0d0-66426de7d63d) - § 1 reference coded [5.22% Coverage]

Reference 1 - 5.22% Coverage

There could be some autistic traits in the family; it can be inherited in short

[<Internals\\Interviews\\INTERVIEW WITH A MEDICAL DOCTOR IN MOMBASA>](../badc1b3b-421d-40ff-b9d0-61a1dffc3343) - § 1 reference coded [1.70% Coverage]

Reference 1 - 1.70% Coverage

Probably it might to the disease…yah. Maybe hereditary

**Treatment options**

**Tradition**

I went to the traditional healer and gave me “vuo” and told me that it was “kiza” for 7 days so I was to wash him with that mixture of herbs and water, and other medication to be applied over the body. I did that one for 7 seven days and went back to the traditional healer and I told him that it did not work and he returned my “fungu”.

Reference 2 - 1.24% Coverage

We used to go to any traditional healer that we heard that he helped someone. They used to treat and my child did not improve, he was given medication for drinking and fumigation but did not improve. When he attained the age of walking and he was not walking, I was directed to a traditional healer by someone, passed Bamba and I had to know the place. When I reached there he was treated. He was put on a stone and was poured with “vuo” and some medication for massaging the legs. They said that my child will walk and I promised that if my child will walk then I will give a sacrifice of a goat. I will slaughter a goat.

[<Internals\\FGDs\\FOCUS GROUP DISCUSSION WITH PARENTS WHO HAVE CHILDREN WITH AUTISM 1>](../86ece053-4338-442d-afcf-2c0150841291) - § 4 references coded [1.01% Coverage]

Reference 1 - 0.14% Coverage

They prayed for me and some were saying that it was a community curse.

Reference 2 - 0.23% Coverage

I told Him “Just look at the whole situation God, you are the one who gave me this being and I cannot do anything”.

Reference 3 - 0.39% Coverage

I have not gone because I am born again and I left everything to Jesus Christ. So I have not gone to a traditional healer because I believe in prayers, and that the prayers will heal my child.

Reference 4 - 0.25% Coverage

For me I would say that they continue praying if they are born again and if they are not saved they accept the condition.

[<Internals\\Interviews\\CONVERSATION BETWEEN GONA AND MAMA AMOS>](../dc187ff3-a46c-44ea-9bcf-2408dfb07374) - § 1 reference coded [1.55% Coverage]

Reference 1 - 1.55% Coverage

Human beings cannot miss something to say, they said and it passed so my prayer is for God to help my child to heal.

[<Internals\\Interviews\\CONVERSATION BETWEEN GONA AND MAMA ISSA>](../45c4b20a-e4a8-4fe7-8ecf-19232074cd7a) - § 1 reference coded [0.85% Coverage]

Reference 1 - 0.85% Coverage

through my child‘s condition I decided to go to church for prayers.

[<Internals\\Interviews\\CONVERSATION BETWEEN GONA AND MAMA LEAH>](../873e28ae-c4d6-47c7-accf-24090ce1ad71) - § 1 reference coded [1.48% Coverage]

Reference 1 - 1.48% Coverage

She went with them, and when they came back the children explained to me that the auntie took them to the pastor and were prayed for the evil spirit sand imitated the pastors.

[<Internals\\Interviews\\CONVERSATION BETWEEN GONA AND MAMA TEDDY>](../39a7b590-b1ad-4cb9-98cf-20ccc4c0c973) - § 2 references coded [1.63% Coverage]

Reference 1 - 0.23% Coverage

I went for prayers.(laughing)

Reference 2 - 1.40% Coverage

I went for prayers and the man of God told me that “this child has no problem it is God who has blessed you with him just like that, we will pray for him and God will perform (miracles)”

[<Internals\\Interviews\\CONVERSATION BETWEEN KEN AND JOHN NGETSA>](../f73aa3d7-f055-48e4-abcf-49acd2f0dc3f) - § 1 reference coded [2.29% Coverage]

Reference 1 - 2.29% Coverage

If these things are given follow up and even the church people get involved, for example a brother to X was prayed for and got healed. If the church can do such good thing, even us (t.h) we can.

**Hospital**

The medications from KEMRI helped my child. The day he first fitted.

[<Internals\\Interviews\\CONVERSATION BETWEEN GONA AND MAMA AMOS>](../dc187ff3-a46c-44ea-9bcf-2408dfb07374) - § 1 reference coded [1.16% Coverage]

Reference 1 - 1.16% Coverage

I have tried a lot in hospitals and there are no places we have not gone in hospitals.

[<Internals\\Interviews\\CONVERSATION BETWEEN GONA AND MAMA ISSA>](../45c4b20a-e4a8-4fe7-8ecf-19232074cd7a) - § 1 reference coded [0.52% Coverage]

Reference 1 - 0.52% Coverage

I only sought treatment from the hospitals

[<Internals\\Interviews\\CONVERSATION BETWEEN GONABABU AND NYANYA LEO>](../c01ad3b4-18b1-4a1f-b3cf-2408f8491f94) - § 1 reference coded [0.16% Coverage]

Reference 1 - 0.16% Coverage

We went to Makadara(Coast General Hospital)

[<Internals\\Interviews\\CONVERSATION BETWEEN GONA AND MAMA ISSA>](../45c4b20a-e4a8-4fe7-8ecf-19232074cd7a) - § 1 reference coded [0.57% Coverage]

Reference 1 - 0.57% Coverage

some efforts of the physiotherapy exercises.

[<Internals\\FGDs\\FOCUS GROUP DISCUSSION WITH PARENTS WHO HAVE CHILDREN WITH AUTISM 1>](../86ece053-4338-442d-afcf-2c0150841291) - § 5 references coded [1.77% Coverage]

Reference 1 - 0.22% Coverage

We took her to hospital and was referred for therapy and after the therapy her behaviors showed some improvement.

Reference 2 - 0.41% Coverage

It took her a long time to attain that, but she could not stand. She continued coming for therapy to help her walk and then was given a walker and stayed for 5 years and that is when he started to walk.

Reference 3 - 0.33% Coverage

We continued with therapy on particular dates and we use to come in all appointments. That continued until he crawled and walked again but he could not speak a word.

Reference 4 - 0.24% Coverage

Yes sometimes we carry the burden then we give up. Then you later come and regret and start again going for therapy. It is good.

Reference 5 - 0.57% Coverage

You will be feeling so bitter then you are the one who gave birth to him/her but you realize that you have gone places and seen others and see that your child is better than the others and that gives you the urge to help your child even more and pray to God to continue helping him/her.

[<Internals\\Interviews\\CONVERSATION BETWEEN GONA AND MAMA ISSA>](../45c4b20a-e4a8-4fe7-8ecf-19232074cd7a) - § 1 reference coded [0.57% Coverage]

Reference 1 - 0.57% Coverage

some efforts of the physiotherapy exercises.

**Prayers**

Reference 1 - 0.14% Coverage

They prayed for me and some were saying that it was a community curse.

Reference 2 - 0.23% Coverage

I told Him “Just look at the whole situation God, you are the one who gave me this being and I cannot do anything”.

Reference 3 - 0.39% Coverage

I have not gone because I am born again and I left everything to Jesus Christ. So I have not gone to a traditional healer because I believe in prayers, and that the prayers will heal my child.

Reference 4 - 0.25% Coverage

For me I would say that they continue praying if they are born again and if they are not saved they accept the condition.

[<Internals\\Interviews\\CONVERSATION BETWEEN GONA AND MAMA ISSA>](../45c4b20a-e4a8-4fe7-8ecf-19232074cd7a) - § 1 reference coded [0.85% Coverage]

Reference 1 - 0.85% Coverage

through my child‘s condition I decided to go to church for prayers.

[<Internals\\Interviews\\CONVERSATION BETWEEN GONA AND MAMA LEAH>](../873e28ae-c4d6-47c7-accf-24090ce1ad71) - § 1 reference coded [1.48% Coverage]

Reference 1 - 1.48% Coverage

She went with them, and when they came back the children explained to me that the auntie took them to the pastor and were prayed for the evil spirit sand imitated the pastors.

[<Internals\\Interviews\\CONVERSATION BETWEEN GONA AND MAMA TEDDY>](../39a7b590-b1ad-4cb9-98cf-20ccc4c0c973) - § 2 references coded [1.63% Coverage]

Reference 1 - 0.23% Coverage

I went for prayers.(laughing)

Reference 2 - 1.40% Coverage

I went for prayers and the man of God told me that “this child has no problem it is God who has blessed you with him just like that, we will pray for him and God will perform (miracles)”

**Treatment expectations**

[<Internals\\FGDs\\FOCUS GROUP DISCUSSION WITH PARENTS WHO HAVE CHILDREN WITH AUTISM 1>](../86ece053-4338-442d-afcf-2c0150841291) - § 1 reference coded [0.68% Coverage]

Reference 1 - 0.68% Coverage

I went and told the teacher, and when I took that step the father was tired because he did not know where to start or where to end as money had gone. I went to Madrassa teacher and told him “ I want my child to be prayed for”(Azunguliwe dua) and asked me if it was to be at the mosque or home and I told him that I wanted it to be at home.

[<Internals\\Interviews\\CONVERSATION BETWEEN GONA AND MAMA FELIX>](../4580088f-1a38-4c40-a9cf-2408dff57b1a) - § 1 reference coded [1.38% Coverage]

Reference 1 - 1.38% Coverage

We would be grateful if doctors would find medication so that we can have hope of these children would come to heal but we do not know what medication will heal these children.

[<Internals\\Interviews\\CONVERSATION BETWEEN GONA AND MAMA TEDDY>](../39a7b590-b1ad-4cb9-98cf-20ccc4c0c973) - § 1 reference coded [1.78% Coverage]

Reference 1 - 1.78% Coverage

What I want most is for the child to be independent as mostly he is dependent of me. If it would be possible for these kinds of children to be independent, be educated and be like the normal children and be able to do things on their own.

[<Internals\\Interviews\\CONVERSATION BETWEEN GONA AND MAMA FELIX>](../4580088f-1a38-4c40-a9cf-2408dff57b1a) - § 2 references coded [2.04% Coverage]

Reference 1 - 1.44% Coverage

I was confused, and was told that this child is not talking so I was to take him to the traditional healer and have his “tongue “cut. I took him and his “tongue” was cut but no improvement.

Reference 2 - 0.60% Coverage

then went to cut his “utasi” so that he could talk but still it did not work.

[<Internals\\Interviews\\CONVERSATION BETWEEN GONA AND MAMA HINZANO>](../d9e52efd-f70a-4790-8fcf-2408e00faa11) - § 2 references coded [3.57% Coverage]

Reference 1 - 0.34% Coverage

I tried the traditional way.

Reference 2 - 3.24% Coverage

I went to traditional healers and he was even “zunguiwa”(a hen swung around his body) believing that it was evil spirits and was also told that it was jins that had made him not talk. I went round until I surrendered because I saw that I was wasting a lot of money and nothing good was coming out
